# Mechanistic analysis for time-dependent effects of cinacalcet on serum calcium, phosphorus, and parathyroid hormone levels in 5/6 nephrectomized rats

**DOI:** 10.1002/phy2.46

**Published:** 2013-08-22

**Authors:** J Ruth Wu-Wong, Masaki Nakane, Yung-wu Chen, Masahide Mizobuchi

**Affiliations:** 1 Department of Pharmacy Practice, University of Illinois at ChicagoChicago, Illinois; 2 VidaGeneChicago, Illinois; 3 Department of Medicine, Showa University School of MedicineTokyo, Japan

**Keywords:** Calcium homeostasis, chronic kidney disease, cinacalcet, Mimpara, parathyroid hormone, Sensipar

## Abstract

This study investigates the time-dependent effects of cinacalcet on serum calcium, phosphorus, and parathyroid hormone (PTH) levels in 5/6 nephrectomized (NX) rats with experimental chronic renal insufficiency. In this study, 5/6 NX male, Sprague–Dawley rats were treated with vehicle or cinacalcet (10 mg/kg, oral, 1× daily). On Day 0 (before treatment), Day 12 and 13 after treatment (to approximate the clinical practice), and also at 0, 1, 4, 8, 16, and 24 hours after the last dosing, blood was collected for analysis. After 12 or 13 days of cinacalcet treatment, modest changes were observed in serum Ca and phosphorus (Pi), while PTH decreased by >45% to Sham levels (152 ± 15 pg/mL). Detailed mapping found that cinacalcet caused a significant time-dependent decrease in serum Ca following dosing, reaching a lowest point at 8 hours (decrease by 20% to 8.43 ± 0.37 mg/dL), and then returning to normal at 24 hours. Cinacalcet also caused a significant increase in serum Pi levels (by 18%). To investigate the potential mechanism of action, a broad approach was taken by testing cinacalcet in a panel of 77 protein-binding assays. Cinacalcet interacted with several channels, transporters, and neurotransmitter receptors, some of which are involved in brain and heart, and may impact Ca homeostasis. Cinacalcet dose-dependently increased brain natriuretic peptide (BNP) mRNA expression by 48% in cardiomyocytes, but had no significant effects on left ventricular hypertrophy and cardiac function. The results suggest that cinacalcet's hypocalcemic effect may be due to its nonspecific interaction with other receptors in brain and heart.

## Introduction

Chronic kidney disease (CKD) patients experience many health issues including mineral and bone disorders and secondary hyperparathyroidism (SHPT) with elevated parathyroid hormone (PTH) (Peacock 2010; Cunningham et al. 2011). One of the mechanisms involved in modulating the secretion of PTH is via changes in the blood Ca^2+^ level, which is sensed by the calcium-sensing receptor (CaSR), a G-protein-coupled receptor, in the parathyroid gland (Brown 2010).

Pharmacological activation of the CaSR can be achieved by ligand binding to two distinct regions of the receptor (Joy et al. 2004). Type I calcimimetics, including inorganic cations and polycatioinic compounds such as neomycin and spermine (Brown et al. 1993; Frazao et al. 2002), bind to the extracellular domain of the CaSR and induce activation in the absence of Ca^2+^. On the other hand, allosteric activators of the CaSR, referred to as type II calcimimetics, activate CaSR through binding to the transmembrane (TM) region, especially TM7, of the receptor and induce a conformational change that increases the receptor's sensitivity to Ca^2+^, resulting in suppression of PTH secretion in the parathyroid gland (Silverberg et al. 1997; Fox et al. 1999; Wada et al. 2000; Goodman et al. 2002). One of the type II calcimimetics, cinacalcet (Mimpara® [Amgen, Thousand Oaks, CA], Sensipar® [Amgen]), has been developed for the treatment of parathyroid carcinoma and hyperparathyroidism secondary to CKD (Block et al. 2004; Dong 2005; Quarles 2005).

Calcium homeostasis is tightly regulated and disturbance to this mechanism lead to hypercalcemia or hypocalcemia, both of which can have important pathological consequences including cardiovascular events (Renkema et al. 2008; Taylor and Bushinsky 2009). Cinacalcet has been linked to hypocalcemia in clinical studies, but the incident is often considered asymptomatic and without significant clinical consequences (Block et al. 2004; Messa et al. 2008; Chonchol et al. 2009). Yet, according to the information provided by the FDA (2012), postmarketing safety surveillance showed that “isolated, idiosyncratic cases of hypotension, and/or worsening heart failure have been reported in patients with impaired cardiac function, in which a causal relationship to Sensipar could not be completely excluded and may be mediated by reductions in serum calcium levels”.

There seems to have inconsistent data about the effect of cinacalcet on serum Ca. According to the package insert for Sensipar (cinacalcet) (Amgen 2012), “serum calcium and serum phosphorus should be measured within 1 week and iPTH should be measured 1–4 weeks after initiation or dose adjustment of Sensipar®. Once the maintenance dose has been established, serum calcium and serum phosphorus should be measured approximately monthly, and PTH every 1–3 months”. Considering the inconsistent clinical data on cinacalcet's effect on serum Ca, there is a need to investigate whether the frequency of monitoring cinacalcet's effect on serum Ca, phosphorus, and PTH in current clinical practice is sufficient or not. For this purpose, this study was designed to map the time-dependent impact of cinacalcet on serum Ca, phosphorus, and PTH in detail in the 5/6 nephrectomized (NX) uremic rats, which develop endothelial dysfunction and left ventricular hypertrophy (Wolf et al. 2000; Gschwend et al. 2002), similar to late-stage CKD patients. A broad hypothesis-generating approach was also taken for follow-up mechanistic analysis in order to investigate why hypocalcemia occurs after cinacalcet treatment.

## Material and Methods

### Subtotally nephrectomized rats

The 5/6 nephrectomy (NX) was performed on male, Sprague–Dawley rats weighing ∼200 g with a standard two-step surgical ablation procedure (Slatopolsky et al. 1995). Rats were maintained on a diet containing 1% calcium and 0.7% phosphorus. Rats at Week 6 after surgery were treated with vehicle (20% hydroxypropyl-β-cyclodextrin, 1.65 mL/kg, p.o. by gavage, once daily) or cinacalcet (10 mg/kg, p.o. by gavage, once daily) for 12 days. On Day 0 (before treatment), Day 12 and 13 after treatment, and at 0, 1, 4, 8, 16, and 24 hours after the last dosing, blood was collected and physiological parameters were measured. To avoid overbleeding any particular rat, each time point was from a different group of rats with a minimum of n = 7 per group. In some experiments, the heart and left ventricle were collected for additional studies.

Separate 5/6 NX rats were treated by cinacalcet (10 mg/kg, p.o. by gavage, once daily for 12 days) for the determination of left ventricular hypertrophy and systolic cardiac function. Cardiac function was determined by the Sonoscape portable color Doppler system, S6V, designed for veterinary application (http://sonoscape.net/EN/products/detail.aspx?id=437,830&&menuId=01,030,205).

The animal studies were conducted under the auspice of Office and Animal Care and Institutional Biosafety, University of Illinois at Chicago. The study conformed with the Guide for the Care and Use of Laboratory Animals published by the US National Institutes of Health (NIH Publication No. 85-23, revised 1996).

### Measurements of physiological parameters

Serum Ca, phosphorus, creatinine, and blood urea nitrogen (BUN) concentrations were measured using a chemistry analyzer (AU400, Olympus, Tokyo, Japan). Serum PTH was measured using a rat intact PTH ELISA kit obtained from ALPCO (Windham, NH).

### Protein panel-binding assays

Binding assays were performed by Cerep (Rueil-Malmaison, France). Primary screening was performed at 10 μmol/L of cinacalcet. Per Cerep's information, a typical protocol for binding assays included a minimum of 6-control wells (nonspecific and total binding) with or without vehicle for the solvent-soluble compound, plus an 8-point dose–response of the relevant reference compound. For some binding assays, two models were available using either agonist or antagonist as the radioligand. The antagonist activity was evaluated by testing increasing concentrations of the compound against a single concentration of the reference agonist. A reference antagonist was used as a positive control. IC_50_ values for the antagonist and maximum responses were then determined (Cerep 2012). Details on the CEREP screen are available from http://www.cerep.fr.

### Cell culture

The rat cardiomyocytes were isolated from 4-day-old rat pups using the Worthington's Neonatal Cardiomyocyte Isolation System. The myocytes were cultured in RPMI Medium 1640 with 10% horse serum, 5% FBS, and 1% antibiotics at 37°C in a humidified 5% CO_2_-95% air atmosphere. Cells were used within 1 week.

### Real-time RT-PCR

Real-time reverse Transcription-polymerase chain reaction (RT-PCR) was performed with an ABI 7500 Fast Real-Time PCR System (Applied Biosystems, Foster City, CA). Each sample had a final volume of 25 μL containing 200 ng of mRNA, 100 nmol/L (final concentration) each of the forward and reverse PCR primers and 250 nmol/L (final concentration) of the TaqMan™ probe (Applied Biosystems). Temperature conditions consisted of a step of 10 minutes at 95°C, followed by 45 cycles of the PCR reaction, and analyzed with the software package (Applied Biosystems). Threshold cycles were determined for each gene.

### Data analysis

Group mean ± SEM are presented. Differences across treatment groups were assessed using a one-way analysis of variance (ANOVA) followed by a Dunnett's post hoc test. A *t*-test was used to assess differences between Day 0 (before treatment) and Day 12 or 13 (after treatment) or as indicated.

## Results

### Serum creatinine and BUN in the 5/6 NX rats

**Figure 1:**
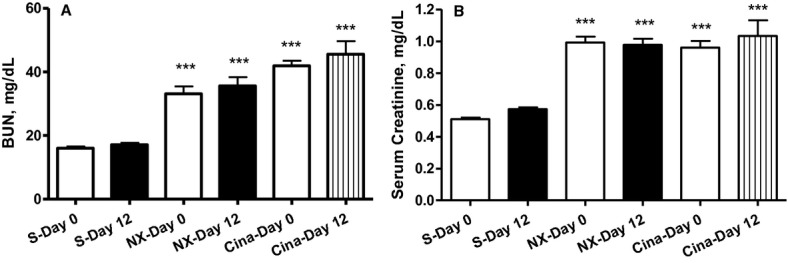
Effects of cinacalcet on serum creatinine and BUN after 12 days of dosing in 5/6 NX rats. SHAM and 5/6 NX rats were treated with vehicle or cinacalcet (10 mg/kg, p.o, 1× daily) for 12 days as described in “Material and Methods”. On Day 0 (before dosing) and Day 12, blood samples were collected for the measurement of BUN (A) and serum creatinine (B). Mean ± standard error was calculated for each group. One-way ANOVA Dunnett test with 95% confidence intervals of difference was performed for statistical comparisons. S, Sham-operate; NX, 5/6 nephrectomized; Cina, cinacalcet. ***P < 0.001 versus Sham-Day 0 (S-Day 0).

As shown in Figure 1, the serum creatinine and blood urea nitrogen (BUN) levels were significantly elevated in 5/6 NX rats compared to Sham rats at 6 weeks after surgery (Sham-Day 0, before drug treatment) thus confirming established renal insufficiency. Treatment with cinacalcet at 10 mg/kg for 12 days did not have an effect on serum creatinine or BUN (Fig. 1A and B).

### Serum calcium in the 5/6 NX rats

**Figure 2:**
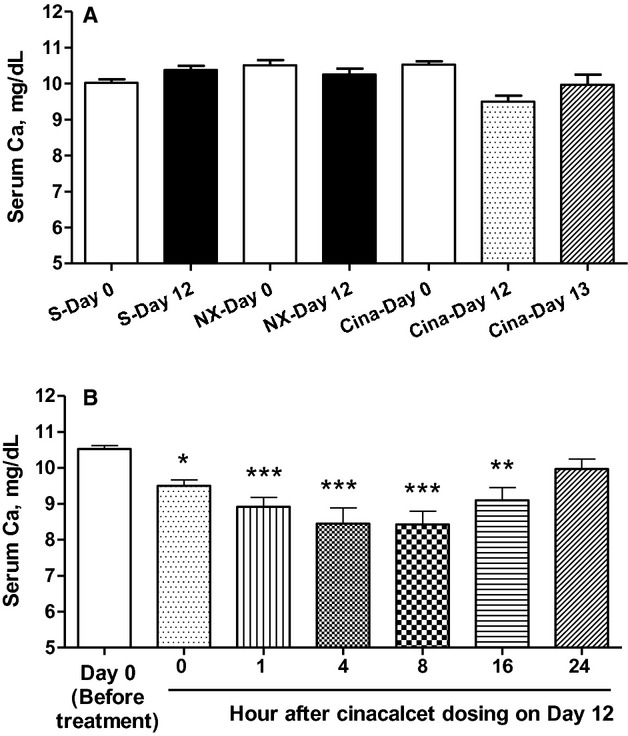
Serum calcium (Ca) levels in 5/6 NX rats. SHAM and 5/6 NX rats were treated with vehicle or cinacalcet (10 mg/kg, p.o, 1× daily) for 12 days as in Figure 1. (A) Blood samples were collected on Day 0 (before dosing) and on Day 12 and Day 13 at 24 hours after dosing, and Ca levels in the serum were measured. Mean ± standard error was calculated for each group. A *t*-test was used to assess differences between Day 0 (before treatment) and Day 12 or 13 (after treatment). One-way ANOVA Dunnett test with 95% confidence intervals of difference was also performed. No statistical significance was detected among the groups. (B) On Day 0 (before treatment) and also on Day 12 at 0, 1, 4, 8, 16, and 24 hours after cinacalcet dosing, blood was collected and Ca levels in the serum were measured. To avoid overbleeding any particular rat, each time point was from a different group of rats with a minimum of n = 7 per group. Mean ± standard error was calculated for each group. One-way ANOVA Dunnett test with 95% confidence intervals of difference was performed for statistical comparisons. *P < 0.05, **P < 0.01, ***P < 0.001 versus Day 0. S, Sham-operate; NX, 5/6 nephrectomized; Cina, cinacalcet.

Figure 2A shows that the serum Ca level was trending lower on Day 12 and Day 13 after 10 mg/kg cinacalcet treatment, but without reaching a statistical significance. However, as shown in Figure 2B, when the effect of cinacalcet on the serum calcium levels was mapped during the 24-hr period after cinacalcet dosing on Day 12, cinacalcet at 10 mg/kg caused a step-wise decrease in serum Ca levels. The serum Ca reached a lowest point at 8 hours after dosing (at 8.43 ± 0.37 mg/dL, a 20% decrease vs. Day 0 at 10.53 ± 0.09 mg/dL), and then gradually went back up and returned to normal (at 9.97 ± 0.28 mg/dL, similar to the level on Day 0 before cinacalcet treatment) at 24 hours after dosing.

### Serum phosphorus in the 5/6 NX rats

**Figure 3:**
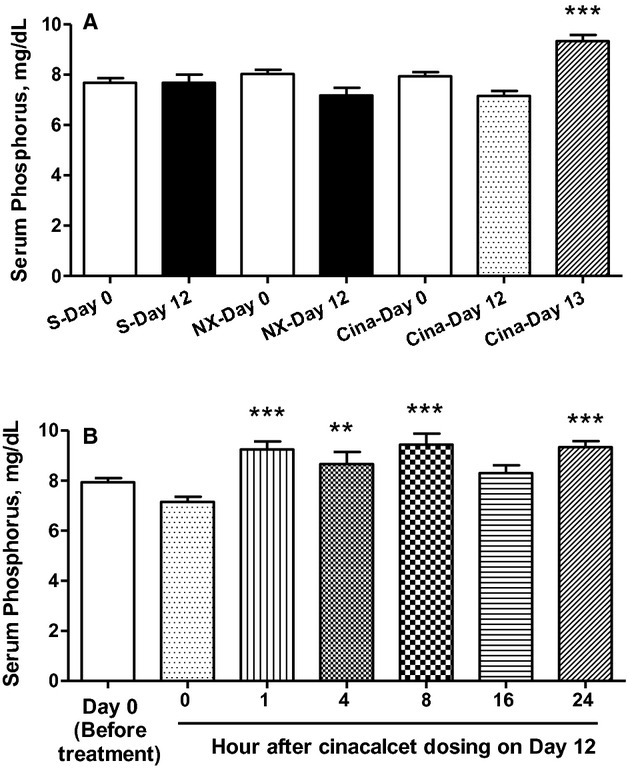
Serum phosphorus (Pi) levels in 5/6 NX rats. SHAM and 5/6 NX rats were treated with vehicle or cinacalcet (10 mg/kg, p.o, 1× daily) for 12 days as in Figure 1. (A) Blood samples were collected on Day 0 (before dosing) and on Day 12 and Day 13 at 24 hours after dosing, and Pi levels in the serum were measured. Mean ± standard error was calculated for each group. A *t*-test was used to assess differences between Day 0 (before treatment) and Day 12 or 13 (after treatment). ***P < 0.001 versus Day 0, same group. (B) On Day 0 (before treatment) and also on Day 12 at 0, 1, 4, 8, 16, and 24 hours after cinacalcet dosing, blood was collected and Pi levels in the serum were measured. Mean ± standard error was calculated for each group. One-way ANOVA Dunnett test with 95% confidence intervals of difference was performed for statistical comparisons. **P < 0.01, ***P < 0.001 versus Day 0. S, Sham-operate; NX, 5/6 nephrectomized; Cina, cinacalcet.

Figure 3A shows that 10 mg/kg cinacalcet treatment for 12 days did not significantly affect serum phosphorus on Day 12, but the serum phosphorus was significantly higher on Day 13. When the effect of cinacalcet on the serum phosphorus levels was mapped during the 24-hour period after cinacalcet dosing (Fig. 3B), cinacalcet at 10 mg/kg caused a significant increase in serum Pi levels observed at 1 hour (at 9.25 ± 0.32 mg/dL, a 16% increase vs. Day 0 at 7.94 ± 0.16 mg/dL), 4 hours (at 8.66 ± 0.48 mg/dL, a 9% increase vs. Day 0), 8 hours (at 9.44 ± 0.44 mg/dL, a 19% increase vs. Day 0), and 24 hours (at 9.34 ± 0.24 mg/dL, a 18% increase vs. Day 0) after dosing, but there was no specific time-dependent pattern in the serum Pi levels after cinacalcet treatment.

### Serum PTH in the 5/6 NX rats

**Figure 4:**
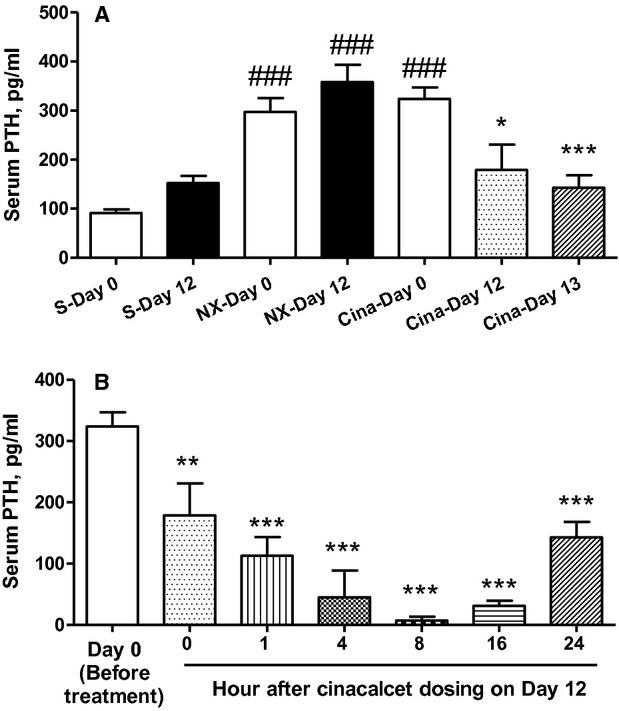
Serum PTH levels in 5/6 NX rats. SHAM and 5/6 NX rats were treated with vehicle or cinacalcet (10 mg/kg, p.o, 1× daily) for 12 days as in Figure 1. (A) Blood samples were collected on Day 0 (before dosing) and on Day 12 and Day 13 at 24 hours after dosing, and PTH levels in the serum were measured. Mean ± standard error was calculated for each group. A *t*-test was used to assess differences between Day 0 (before treatment) and Day 12 or 13 (after treatment). *P < 0.05, ***P < 0.001 versus Day 0, same group. One-way ANOVA Dunnett test with 95% confidence intervals of difference was also performed. ###*P* < 0.001 versus Sham on Day 0. (B) On Day 0 (before treatment) and also on Day 12 at 0, 1, 4, 8, 16, and 24 hours after cinacalcet dosing, blood was collected and PTH levels in the serum were measured. Mean ± standard error was calculated for each group. One-way ANOVA Dunnett test with 95% confidence intervals of difference was performed for statistical comparisons. **P < 0.01, ***P < 0.001 versus Day 0. S, Sham-operate; NX, 5/6 nephrectomized; Cina, cinacalcet.

Figure 4A shows that the serum PTH levels were significantly elevated in the NX rats (297.3 ± 28.2 pg/mL vs. Sham-Day 0 at 90.9 ± 7.7 pg/mL). Cinacalcet treatment (10 mg/kg) for 12 days reduced the PTH level by >45% on Day 12 and Day 13 (vs. Cina-Day 0). When the effect of cinacalcet on the serum PTH levels was mapped during the 24-hour period after cinacalcet dosing (Fig. 4B), cinacalcet at 10 mg/kg caused a step-wise decrease in serum PTH levels, reaching a lowest point (PTH = 7.2 ±6.2 pg/mL) at 8 hours after dosing before returning to a level (142.7 ± 25.4 pg/mL at 24 hours) similar to that at Time 0 on Day 12 (178.8 ± 52.0 pg/mL).

### Correlation among serum Ca, phosphorus, and PTH in the 5/6 NX rats

**Figure 5:**
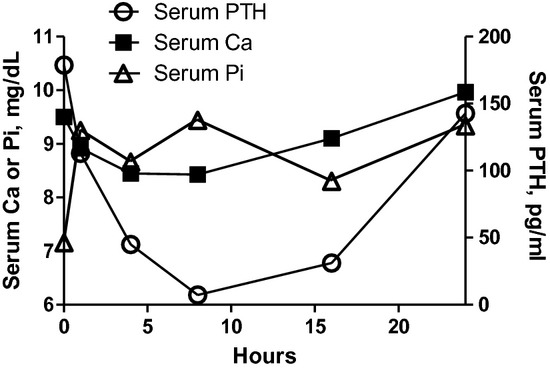
Correlation among serum Ca, Pi, and PTH. The serum Ca, Pi, and PTH values from Day 12 at 0, 1, 4, 8, 16, and 24 hours after cinacalcet dosing were plotted together to investigate the potential correlation among the three parameters.

It is generally thought that the hypocalcemic effect of cinacalcet is the result of the suppression of PTH and/or the increase in serum phosphorus. However, when the 3 parameters were plotted together as shown in Figure 5, there was no clear correlation pattern between serum Ca and phosphorus, or between serum PTH and Ca, suggesting that factors other than parathyroid hormone and/or serum phosphorus may contribute to cinacalcet's effect on serum Ca.

### Binding of cinacalcet to channels, transporters and neurotransmitter receptors

**Figure 6:**
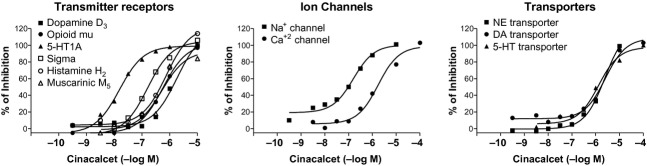
Binding of cinacalcet to receptors, ion channels, and transporters. Binding studies were conducted as described in Material and Methods. Briefly, increasing concentrations of cinacalcet were tested against a single concentration of the reference agonist. A reference antagonist is used as a positive control. IC_50_ values for the antagonist and maximum responses were determined.

How cinacalcet causes hypocalcemia is not well understood. For hypothesis-generating purposes, we took a broad approach by testing cinacalcet in a panel of 77 protein-binding assays in order to develop new understanding of cinacalcet's effects. While cinacalcet has no effect on a majority of proteins, it affects the binding of native ligand to several ion channels, transporters, and neurotransmitter receptors. The IC_50_ determination studies show that cinacalcet affected the binding of native ligand to mu-opiate receptor (opioid mu), 5-hydroxytryptamine1A receptor (5-HT1A), haloperidol-sensitive sigma sites (sigma), and Na^+^ channel in the sub-μmol/L range (Fig. 6). Cinacalcet affected the binding of native ligand to dopamine receptor (dopamine D_3_), noradrenaline (NE) transporter, dopamine (DA) transporter, histamine H_2_ receptor (histamine H_2_), muscarinic receptor (muscarinic M5), Ca^2+^ channel, and 5-hydroxytryptamine (5-HT) transporter in the μmol/L range (Fig. 6). The IC_50_ results are summarized in Table 1.

**Table 1:** Summary of cinacalcet's IC_50_ values from binding studies for selected proteins

Target protein	IC_50_, μmol/L
D_3_ (h): dopamine receptor	1.8
H_2_ (h): histamine H_2_ receptor	7.3
M_5_ (h): muscarinic receptor	5.1
μ-opiate receptor (h) (agonist site)	0.54
5-HT1A (h): 5-hydroxytryptamine1A (5-HT1A) receptor	0.015
Sigma: haloperidol-sensitive sigma sites in the striatum, hippocampus and cerebral cortex	0.15
Ca^2+^ channel (L, verapamil site) (phenylalkylamines)	4.8
Na^+^ channel (site 2)	0.15
NE transporter (h): noradrenaline transporter	2.3
DA transporter (h): dopamine transporter	3.8
5-HT transporter (h): 5-hydroxytryptamine (serotonin) transporter	3.8

### Effects on brain natriuretic peptide mRNA expression in cardiomyocytes

**Figure 7:**
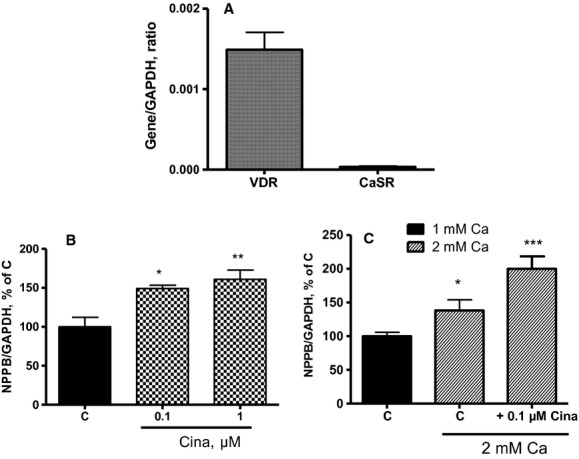
Effects of cinacalcet on NPPB expression in neonatal rat cardiomyocytes. (A): Neonatal rat cardiomyocytes were harvested, RNA isolated and the VDR or CaSR mRNA level analyzed by real-time RT-PCR. GAPDH was used for normalization. (B) and (C): Neonatal rat cardiomyocytes in culture medium containing 1 mmol/L Ca (B) or 2 mmol/L Ca (C) were treated with or without cinacalcet (concentrations as indicated) for 24 hours. Cells were harvested, RNA isolated, and the NPPB mRNA level analyzed by real-time RT-PCR. The NPPB level was first normalized with GAPDH, and then expressed as% of control (C: no drug). Statistical analysis was performed by one-way ANOVA followed by a Dunnett's post hoc test. *P < 0.05, **P < 0.01, ***P < 0.001 compared with control (no treatment); n = 4 per condition.

Because cinacalcet binds to several proteins that are involved in the cardiovascular system, follow-up studies using neonatal rat cardiomyocytes were also conducted. NPPB encodes brain natriuretic peptide (BNP), which is a biomarker for cardiovascular disease (Di Angelantonio et al. 2009; Ritchie et al. 2009). Figure 7A shows that the cardiomyocytes expressed a very low level of CaSR (vs. VDR as the positive control) in the cardiomyocytes. Figure 7B shows that cinacalcet at 0.1 and 1 μmol/L elevated BNP mRNA expression by 48 and 60% in these cells in normal culture medium containing 1 mmol/L Ca. Figure 7C shows that elevating the Ca concentration to 2 mmol/L in the culture medium upregulated the expression of BNP mRNA by 38%, and cinacalcet (0.1 μmol/L) plus 2 mmol/L Ca further elevated BNP mRNA by an additional 62%.

### Effects on left ventricular hypertrophy and cardiac function in the 5/6 NX rats

**Figure 8:**
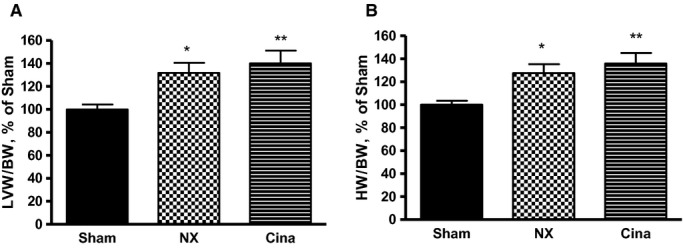
Left ventricular hypertrophy in 5/6 NX rats and the effect of cinacalcet. Sham and 5/6 NX rats were treated with vehicle or cinacalcet (10 mg/kg, p.o, 1× daily) for 12 days as described in “Material and Methods”. Heart was collected and weighed. Left ventricle (LV) was then dissected and weighed. LV (A) or heart (B) weight was first normalized by body weight and then expressed as% of control (Sham). Group mean ± SEM are presented. One-way ANOVA Dunnett test with 95% confidence intervals of difference was performed for statistical comparisons. *P < 0.05, **P < 0.01 versus Sham. LVW, LV weight; HW, heart weight; BW, body weight.

We then examined the effects of cinacalcet on left ventricular hypertrophy (LVH). The 5/6 NX rats are known to develop LVH (Wolf et al. 2000). Figure 8A shows that at 8 weeks after the renal ablation surgery, the left ventricle weight (LVW) versus body weight (BW) ratio as a percentage of control was significantly higher (32% increase) in the 5/6 NX rats (vs. sham). Another control study found that LVH was present in the 5/6 NX rats at 6 weeks after the second surgery (data not shown). Figure 8A demonstrates that 2-week treatment with cinacalcet at 10 mg/kg (oral, 1× daily) did not reduce the LVW/BW ratio. Figure 8B shows a similar observation for the heart weight (HW) versus BW ratio. The LV systolic function data were summarized in Table 2. Consistent with the LVW/BW data, 2-week treatment with cinacalcet at 10 mg/kg (oral, 1× daily) did not significantly change the LV systolic function in the 5/6 NX rats.

**Table 2:** Summary of cinacalcet's effect on left ventricular systolic function

Parameters	5/6 NX-vehicle (n = 6)	5/6 NX-cinacalcet (n = 7)
Left ventricular end-diastolic dimension, cm	0.60 ± 0.06	0.57 ± 0.06
Left ventricular end-systolic dimension, cm	0.29 ± 0.04	0.29 ± 0.05
Fractional shortening,%	51.7 ± 3.6	47.4 ± 10.6
Left ventricular end-diastolic volume, mL	0.18 ± 0.04	0.16 ± 0.04
Left ventricular end-systolic volume, mL	0.03 ± 0.01	0.03 ± 0.01
Ejection fraction,%	81.9 ± 3.37	76.7 ± 11.1
LV mass index, mg/g	1.31 ± 0.25	1.28 ± 0.36

Rats were treated with vehicle or cinacalcet as in Figure 8. Data presented are Mean ± SEM. *t*-Test was used to assess differences between the two groups: no statistical significance was detected for any of the parameters.

## Discussion

This study was to map the time-dependent impact of cinacalcet on serum Ca in order to understand why there are inconsistent data about the effect of cinacalcet on serum Ca from different clinical studies. For example, previously it was demonstrated that in a clinical study testing cinacalcet versus placebo for 26 weeks in patients on maintenance dialysis, mean PTH values decreased by 43% in the cinacalcet group, but increased by 9% in the placebo group, and there was a modest reduction in serum Ca (Block et al. 2004). Messa et al.(2008) reported from the OPTIMA study that cinacalcet ranging from 30 to 180 mg once daily resulted in 71% of patients achieving an iPTH level at ≤300 pg/mL, while mean serum Ca decreased by 7%. In a study testing cinacalcet versus placebo in Stage 3 or 4 CKD patients not on dialysis for 32 weeks, Chonchol et al. (2009) reported that cinacalcet ranging from 30 to 180 mg once daily resulted in 74% of the patients achieving a > 30% decrease in iPTH. Cinacalcet decreased serum Ca by 12% at Week 16 and 10% at Week 32. The study also reported that cinacalcet tended to increase serum phosphorus. Padhi et al. (2012) reported that in pediatric patients undergoing hemodialysis, hypocalcemia was detected in 50% of the patients after one single dose of cinacalcet (15 mg). The study also mapped mean serum Ca concentrations by time for each age-cohort, and found that the maximum decrease in serum Ca was observed at around ∼8 hours after dosing, and the serum Ca level returned to the baseline level at 24 hours after dosing. However, the effect of cinacalcet on serum intact PTH (iPTH) concentrations was only observed at 2-hour postdosing in most patients. Recently, Ketteler et al. (2012) reported the results from the IMPACT study that hypocalcaemia occurred in ∼50% of cinacalcet-treated subjects.

Results from this study provide a plausible explanation for the inconsistent results of cincalcet's effect on serum Ca. Data from this current study show that cinacalcet after 12 days of treatment has a modest effect on serum Ca and Pi, while effectively reduces serum PTH by 45% to the Sham level. However, by measuring serum Ca and Pi every few hours following cinacalcet dosing, the results indicate that cinacalcet increases serum Pi, and causes a significant step-wise reduction in serum Ca and PTH, reaching a maximal effect at ∼8 hours after dosing. It seems that different conclusions may be drawn regarding the effect of cinacalcet on serum Ca and Pi depending on when blood samples are collected for analysis after cinacalcet dosing. Since the various clinical studies might not have used the same scheduling to collect blood samples, perhaps it is not surprising that different results and conclusions regarding cinacalcet's effect on serum Ca were reached.

Since we did not see a clear correlation between serum Ca and PTH (and/or phosphorus), a broad, hypothesis-generating approach was taken to investigate the mechanism of action for cinacalcet's effect on serum Ca. It is a surprise to find that cinacalcet interacts with neurotransmitter receptors including the dopamine receptor and the 5-hydroxytryptamine1A (5-HT1A) receptor. While the next level of investigation should involve binding studies using radiolabeled cinacalcet, we note that previously it has been shown that compounds that interact with neurotransmitter receptors may affect synthesis of calcium-binding proteins in the tissues, and alter calcium-dependent signaling pathways, which will affect calcium homeostasis and lead to a calcium-depleted state and/or trigger calcium redistribution within body compartments (el-Defrawi and Craig 1984; MacDonald et al. 2005; Rushlow et al. 2009). Risperidone, an atypical neuroleptic that antagonizes 5-HT1A and also different isoforms of dopamine receptors, has been associated with hypocalcemia (Smith et al. 2006; Schatzberg and Nemeroff 2009; Milovanovic et al. 2010). Our results provide an alternative explanation why cinacalcet causes hypocalcemia.

The broad, hypothesis-generating approach also uncovered that cinacalcet interacts with channels such as Na^+^ channel and Ca^2+^ channel, which are known to play roles in the cardiovascular system. Follow-up functional studies show that neonatal rat cardiomyocytes express a very low level of CaSR, yet cinacalcet does affect the expression of BNP mRNA significantly at either 1 or 2 mmol/L Ca. Although it cannot be ruled out that the low level of CaSR expression in cardiomyocytes is responsible for cinacalcet's effect on BNP mRNA expression, the interaction between cinacalcet and the other proteins can also lead to its effect on BNP expression as reported previously that some of these proteins are involved in regulating BNP expression (Lubic et al. 1995; Kudoh et al. 2003; Hall 2005). BNP is known to be associated with LVH (Cosson 2004; Ritchie et al. 2009), a common condition in CKD, which often leads to heart failure (Bluemke et al. 2008) and increased risks of hospitalization and mortality (Gwadry-Sridhar et al. 2004; Sciacqua et al. 2006; Pons et al. 2010). The 5/6 nephrectomized (NX) uremic rats, similar to late-stage CKD patients, develop endothelial dysfunction and LVH (Wolf et al. 2000; Gschwend et al. 2002). Our data show that cinacalcet does not have a significant effect on the LVW/BW and HW/BW ratios in the 5/6 NX rats, and also did not affect LV systolic function significantly. Our observations are consistent with the results from the recent clinical study (EVOLVE) that hypocalcemia and gastrointestinal adverse events were significantly more frequent in patients receiving cinacalcet, while cinacalcet did not significantly reduce the risk of death or most of cardiovascular events in dialysis patients with moderate-to-severe SHPT (Carney 2012; Chertow et al. 2012).

One limitation of this study is the lack of data on the FGF23 status before and after cinacalcet treatment. FGF23, a phosphorus regulating factor (Wolf 2010), has gained significance in the CKD field during the past decade. Excessive FGF23 levels, which increase progressively beginning in early stages of kidney disease in order to maintain normophosphatemia despite decreased nephron mass, may be partially responsible for early calcitriol deficiency and secondary hyperparathyroidism in CKD (Gutierrez et al. 2005). Our data suggested that cinacalcet exhibits effects on serum phosphorus, which may lead to changes in serum FGF23 levels. It has been shown that in 55 hemodialysis patients after 12 weeks of cinacalcet treatment, FGF23 levels decreased significantly concomitantly with a significant reduction in PTH levels (Koizumi et al. 2011). A similar observation was made in 91 subjects over the course of the ACHIEVE trial that treatment with cinacalcet plus low-dose calcitriol analogs resulted in lower FGF23 levels (Wetmore et al. 2010). A study done in the 5/6 NX rats also showed that cinacalcet promoted hypocalcemia and marked hyperphosphatemia, but serum FGF23 tended to decrease (Finch et al. 2010).

This study testing cinacalcet at 10 mg/kg with samples collected on Day 12 or Day 13 after treatment was intended to approximate the clinical practice, and the data fail to show consistent effects of cinacalcet on serum Ca and Pi. Only when samples are collected every few hours following cinacalcet dosing, the effects of cinacalcet on serum Ca, Pi, and PTH are more accurately revealed. Although this study was conducted in an experimental chronic renal insufficiency animal model, our data suggest that the inconsistent observations in clinical settings about the effect of cinacalcet on serum Ca may be partially associated with the current clinical practice in monitoring the Ca parameter, which perhaps is not frequent enough. Our results also suggest that cinacalcet may incur long-term effects on brain and heart, which will require additional studies.
